# Data highlighting miR-155 and GAPDH correlation

**DOI:** 10.1016/j.dib.2019.103945

**Published:** 2019-04-27

**Authors:** Madhu Khanna, Sanjesh Saini, Malini Shariff, Larance Ronsard, Jitender K. Singh, Harish Kumar

**Affiliations:** a Vallabhbhai Patel Chest Institute, University of Delhi, 110007, India; b Ragon Institute, USA

**Keywords:** MiR-155, Glucose metabolism, Housekeeping genes, microRNA electroporation

## Abstract

This data represents the effect of miR-155 on the expression of commonly used housekeeping genes, GAPDH, Beta Actin, RPL13A, and U6. The human miR-155 and control RNA were transfected to A549 cells by electroporation. Expression of these genes was compared in both groups by real-time PCR. The significant up-regulation in the expression of GAPDH was observed in the miR-155 transfected samples as compared to control while no major change was observed in the expression of the other three genes.

**Table:** Specifications table

Subject area	Biology
More specific subject area	Molecular biology
Type of data	Tables and figures
How data was acquired	Real-time PCR data acquired and analyzed by CFX manager software
Data format	Analyzed
Experimental factors	MiR-155 (30nM), time (24 hour post-transfection)
Experimental features	A549 cells were transfected with microRNA, and differential gene expression was analyzed
Data source location	Vallabhbhai Patel Chest Institute, University of Delhi, Delhi, India
Data accessibility	Data presented in current article only
Related research article	Kim S, Lee E, Jung J, Lee JW, Kim HJ, Kim J et al. microRNA-155 positively regulates glucose metabolism via PIK3R1-FOXO3a-cMYC axis in breast cancer. Oncogene. 2018 May; 37 (22):2982–91. [1]

**Table:** 

**Value of the data** • The data represents a correlation between miR-155 and GAPDH expression. Since the GAPDH is an essential enzyme of the glycolysis pathway, therefore the association of miR-155 with glycolytic pathways may be explored. • The data will help in choosing better housekeeping genes for miR-155 transfection experiments. • The data is valuable for those working on microRNA electroporation.

## 1 Data

**Fig. 1:**
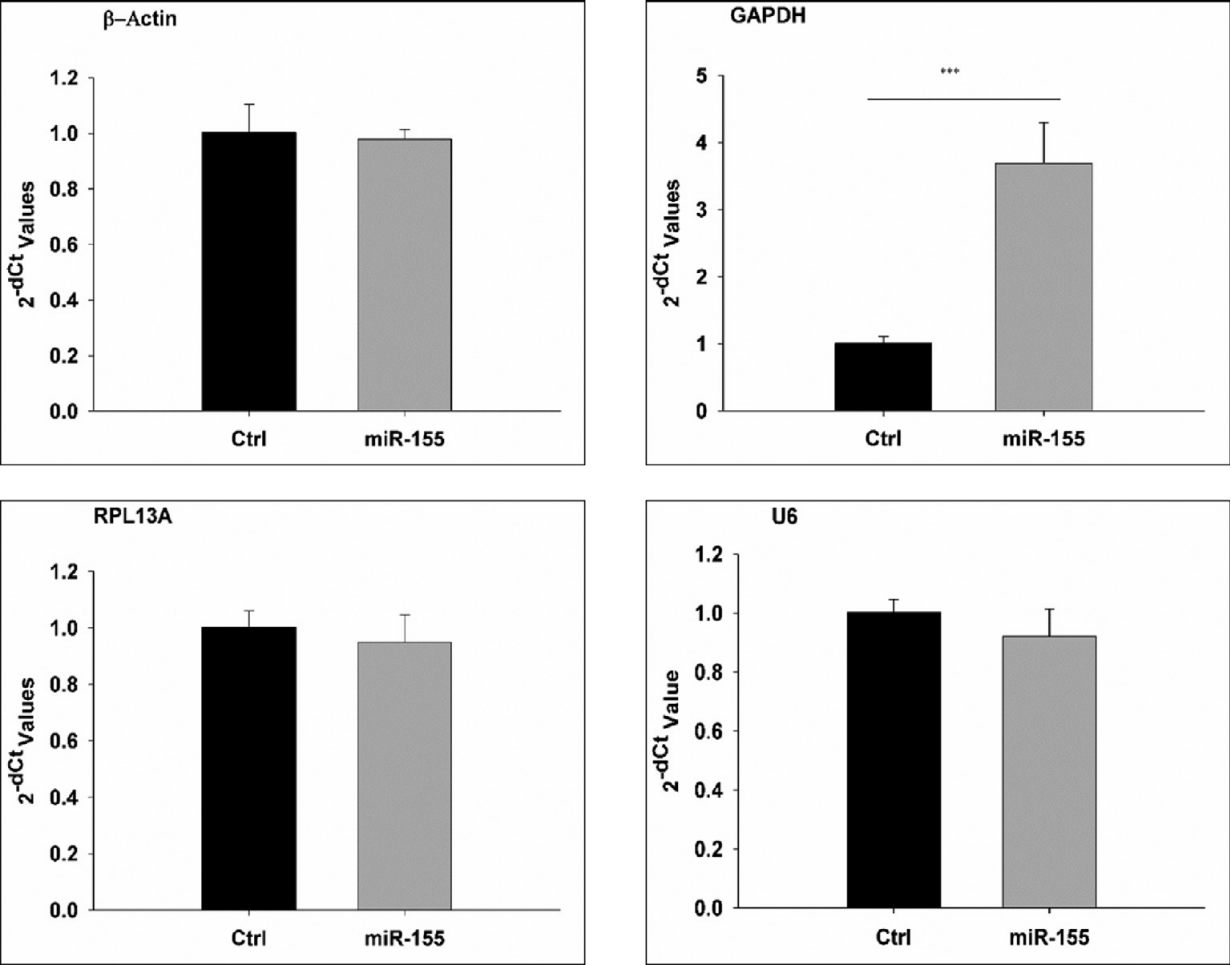
The $2^{-\Delta \text{Ct}}$ value of genes compared in the miR-155 transfected and control group (ctrl). (***p value < 0.001).

We report the relative gene-expression profile of four genes, Glyceraldehyde-3-Phosphate Dehydrogenase (GAPDH), Beta-actin (β-Actin), Ribosomal Protein L13a (RPL13A) and U6 snRNA, in response to a microRNA (miR-155). The candidate cells, A549 cell line, were electroporated with miR-155 and Negative control RNA. The total RNA was isolated 24-h post-transfection and cDNA was prepared. We compared the expression profile of each gene at the transcriptomic level by quantitative-Polymerase Chain Reaction (qPCR) method. The qPCR efficiency, represented in Table 1, of each primer, was assessed by making the standard curve. The expression profiling of all four genes in control and miR-155 transfected samples shows a significant (p < 0.001) difference in the expression of gene GAPDH as compared to other test genes (Table 2). The data presented in Table 2 is Ct value data of qPCR performed on the cDNA sample obtained from A549 cells transfected with miR-155 and the cell transfected with negative control RNA. The Ct value representing in Table 2 is the average of technical replicates for each sample. Fig. 1 shows the upregulation in the expression of gene GAPDH in the miR-155 transfected group as compared to the control group while no critical change observed in the expression of other genes. The experiment performed on three biological and three technical replicates of each sample using primers in Table 3.

**Table 1:** **qPCR efficiency of target genes**. 10 fold serial dilution of the target template of each primer was prepared, and real-time PCR was performed to calculate the qPCR efficiency.

Target Gene	qPCR Efficiency
GAPDH	95%
β-Actin	98%
RPL13A	93%
U6 snRNA	High efficiency claimed by manufacturer (ThermoScientific)

**Table 2:** Ct value data.

Gene	Ct Value (Control)	Ct Value (miR-155 transfected)	p-value (based on $2^{-\Delta \text{Ct}}$)
GAPDH	20.18	18.19	0.0008
	20.48	18.61	
	20.31	18.56	
β-Actin	25.53	25.71	0.3609
	25.82	25.76	
	25.69	25.66	
U6	22.29	22.33	0.2315
	22.42	22.61	
	22.37	22.51	
RPL13A	19.35	19.20	0.1246
	19.21	19.50	
	19.19	19.30

**Table 3:** Primer sequences.

Primer	Sequence	Reference
GAPDH (Glyceraldehyde 3-phosphate dehydrogenase)	Forward:TGCACCACCAACTGCTTAGC	[2]
	Reverse: GGCATGGACTGTGGTCATGAG	
RPL13A	Forward: GCCCTACGACAAGAAAAAGCG	[3]
Ribosomal Protein L13a	Reverse: TACTTCCAGCCAACCTCGTGA	
Beta Actin	Forward: GTCTGCCTTGGTAGTGGATAATG	[4]
	Reverse: TCGAGGACGCCCTATCATGG	
U6 snRNA	Predesigned primer probe set ((assay ID 001973, Thermo scientific)	

## 2 Experimental design, material and methods

### 2.1 Culture of human alveolar epithelial cell lineA549

Human alveolar epithelial cell line (A549) was maintained in Dulbecco's Modified Eagle Medium (DMEM) containing 10% fetal bovine serum (FBS), 100 units/ml penicillin, and 100 units/ml streptomycin in tissue culture ﬂasks at 37 °C with 5% CO_2._

### 2.2 microRNA transfection

A549 cells pellet was washed with 1XPBS and resuspended in electroporation buffer (Catalog # 1652676, Bio-Rad) with final cell number 1 × 10^6^ cells/ml. The final concentration of microRNA (hsa-miR-155–5p mimic (Catalog # 4464066, Assay ID MC12601, Thermo scientific), Negative control microRNA (Catalog# 4464077, Thermo Scientific)) in electroporation buffer was 30nM. Electroporation was performed in 0.2ml Cuvette at 150V for 10 ms in a single pulse. Cells were seeded in six well plate after electroporation.

### 2.3 RNA isolation, cDNA preparation and real-time PCR

Total RNA was isolated using miRVANA RNA isolation kit (Catalog# AM1560, Thermo scientific) as per manufacturer's instructions. RNA quantification was done by qubit 3.0 fluorometer (Catalog #Q33216, life technologies). Total RNA (1μg) was reverse transcribed using high capacity reverse transcription kit (Catalog# 4368814, Thermo scientific) with oligo dT primer. PCR reaction (20μl) for the genes GAPDH, Beta Actin and RPL13A was performed using 2 μl of cDNA, 10 μlSSo Fast Eva green (Catalog# 1725203, Bio-Rad) and 0.25pmol primer mix (forward and reverse) on CFX96 Touch real-time PCR system (Catalog# 1855195, Bio-Rad) with temperature conditions as follow: Initial denaturation at 98 °C for 2min followed by 30 cycles of 98 °C for 5 sec and 60 °C for 5 sec.

The reverse transcription and PCR reaction of U6 was performed using predesigned assay (assay ID 001973, Thermo Scientific) as per the manufacturer instruction. Briefly, 10ng total RNA was reverse transcribed using RT primer with TaqMan reverse transcription kit (Catalog# 4366596, Applied Biosystems). PCR reaction was performed using a specific Taq Man primer-probe set by PCR master mix (Catalog# 4324018, Applied Biosystems).

The Ct value data was collected by CFX manager software (Bio-rad).

$2^{-\Delta \text{Ct}}$ calculations were as follow:

$$2^{-\Delta \text{Ct}}=2^{-\left(\text{average}\;\text{Ct}\;\text{of}\;\text{Contol}-\text{Ct}\;\text{of}\;\text{individual}\;\text{sample}\right)}$$

### 2.4 Primer efficiency calculation

PCR reaction was performed for each gene of Table 1, using 2 μl cDNA. The PCR product of each gene was run on the agarose gel, DNA band was excised from the agarose gel and purified using gel extraction kit (Catalog# 28115, Qiagen) as per the manufacturer's instructions. The 10-fold serial dilution of all purified DNA samples was prepared and amplified by real-time PCR using the specific primer to generate a standard curve. Primer efficiency was calculated from the slop using formula.

$$\text{E}=10^{-\left(1/\text{slope}\right)}-1$$

### 2.5 Statistical analysis

Unpaired T-test was applied on the $2^{-\Delta \text{Ct}}$ value of the data obtained from the samples transfected with negative control and miR-155.
