# Supplementary material for: Data highlighting miR-155 and GAPDH correlation
Source: Data Brief. 2019 Apr 27;24:103945. doi: 10.1016/j.dib.2019.103945 (PMC6523032; doi:10.1016/j.dib.2019.103945)
Supplement: Multimedia component 1 [file mmc1.pdf]

Conflicts of Interest Statement

Manuscript title: Data Highlighting miR-155 and GAPDH

The authors whose names are listed immediately below certify that they have NO affiliations with or involvement in any organization or entity with any financial interest (such as honoraria; educational grants; participation in speakers' bureaus; membership, employment, consultancies, stock ownership, or other equity interest; and expert testimony or patent-licensing arrangements), or non-financial interest (such as personal or professional relationships, affiliations, knowledge or beliefs) in the subject matter or materials discussed in this manuscript.

Author names:

Prof. Madhu Khanna
Prof. Malini Shariff
Mr. Sanjesh Saini
Mr. Larance Ronsard
Mr. Jitender K. Singh
Mr. Harish Kumar

The authors whose names are listed immediately below report the following details of affiliation or involvement in an organization or entity with a financial or non-financial interest in the subject matter or materials discussed in this manuscript. Please specify the nature of the conflict on a separate sheet of paper if the space below is inadequate.

Author names:
